# Epigenetic Tailoring for the Production of Anti-Infective Cytosporones from the Marine Fungus *Leucostoma persoonii*

**DOI:** 10.3390/md10040762

**Published:** 2012-03-28

**Authors:** Jeremy Beau, Nida Mahid, Whittney N. Burda, Lacey Harrington, Lindsey N. Shaw, Tina Mutka, Dennis E. Kyle, Betty Barisic, Alberto van Olphen, Bill J. Baker

**Affiliations:** 1 Department of Chemistry and Center for Molecular Diversity in Drug Design, Discovery and Delivery, University of South Florida, Tampa, FL 33620, USA; Email: jbeau@mail.usf.edu (J.B.); nmahid@mail.usf.edu (N.M.); 2 Department of Cell Biology, Microbiology and Molecular Biology, University of South Florida, Tampa, FL 33620, USA; Email: wburda@mail.usf.edu (W.N.B.); laharrin@mail.usf.edu (L.H.); shaw@usf.edu (L.N.S.); 3 Department of Global Health, University of South Florida, Tampa, FL 33620, USA; Email: tmutka@health.usf.edu (T.M.); dkyle@health.usf.edu (D.E.K.); bbarisic@health.usf.edu (B.B.); avanolph@health.usf.edu (A.O.)

**Keywords:** epigenetics, fungus, mangrove, MRSA, malaria

## Abstract

Recent genomic studies have demonstrated that fungi can possess gene clusters encoding for the production of previously unobserved secondary metabolites. Activation of these attenuated or silenced genes to obtain either improved titers of known compounds or new ones altogether has been a subject of considerable interest. In our efforts to discover new chemotypes that are effective against infectious diseases, including malaria and methicillin-resistant *Staphylococcus aureus* (MRSA), we have isolated a strain of marine fungus, *Leucostoma persoonii*, that produces bioactive cytosporones. Epigenetic modifiers employed to activate secondary metabolite genes resulted in enhanced production of known cytosporones B (**1**, 360%), C (**2**, 580%) and E (**3**, 890%), as well as the production of the previously undescribed cytosporone R (**4**). Cytosporone E was the most bioactive, displaying an IC_90_ of 13 µM toward *Plasmodium falciparum*, with A549 cytotoxicity IC_90_ of 437 µM, representing a 90% inhibition therapeutic index (TI_90_ = IC_90_ A459/IC_90_ *P. falciparum*) of 33. In addition, cytosporone E was active against MRSA with a minimal inhibitory concentration (MIC) of 72 µM and inhibition of MRSA biofilm at roughly half that value (minimum biofilm eradication counts, MBEC90, was found to be 39 µM).

## 1. Introduction

**Figure 1:**
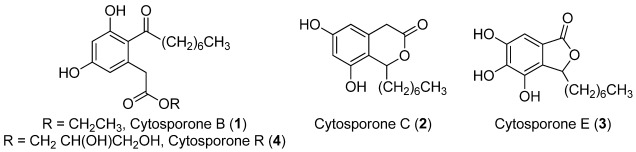
Structures of the cytosporones (**1**–**4**).

After coral reefs, mangrove communities are the most important ecosystem in the marine environment in terms of productivity and sustained tertiary yield. A study of over 150 species of fish demonstrated that the coral reefs near mangroves contained on average twice as much biomass as the reefs not near this niche [1]. The mangrove microenvironment is demanding of plants, as well as associated organisms living on or within their tissues, due to, for example, frequent variation of salinity, temperature and moisture. In addition, predation pressure is intense in these high-biodiversity systems. Mangrove tissues are replete with endophytic bacteria and fungi, and chemical investigations of these microbes have documented diverse bioactive natural products [2,3,4,5,6,7,8,9,10]. In our efforts to isolate antimalarial compounds from Floridian mangrove endophytes, we isolated a strain of the endophytic fungus *Leucostoma persoonii* from red mangrove, *Rhizophora mangle*, that showed moderate activity against malaria. We subsequently tested the extract against a variety of disease targets and found promising activity against a highly resistant methicillin-resistant *Staphylococcus aureus* (MRSA). Based on recent results using epigenetic modifications in fungi to produce new bioactive compounds [11,12,13], we experimented with small molecule epigenetic modifiers to enhance production of secondary metabolites in our fungal isolate. We report here conditions for the production of enhanced levels of known cytosporones B (**1**), C (**2**), and E (**3**), as well as a previously uncharacterized cytosporone [14,15,16,17] cytosporone R (**4**) (Figure 1). 

## 2. Results and Discussion

### 2.1. Fungal Isolation and Identification

*Leucostoma persoonii* was isolated from red mangrove, *Rhizophora mangle*, branch tissues obtained from the Florida Everglades in the summer of 2010. In the field, a portion of the branch was surface sterilized then shavings of the bark were placed onto saline cornmeal agar. The plates were incubated for 3 days prior to colony selection and purification of fungal isolates. *L. persoonii* was found as a slow growing tan/yellow filament. Analysis of the 18S ribosome DNA by BLAST database screening provided a 99% match to *L. persoonii*.

### 2.2. Malaria Screening

Mangrove endophytes isolated as described above were subject to small-scale cultivation on Sabouraud dextrose agar (SDA), freeze dried, then extracted with methanol. Extracts at a concentration of 30 mg/mL were screened in a luciferase-reporter *Plasmodium falciparum* assay [18]. The *L. persoonii* extract demonstrated >50% inhibition at 50 µg/mL and was therefore selected for scale-up cultivation and bioassay-guided isolation studies.

### 2.3. Antibiotic Screening

Concomitant with malaria screening, aliquots of fungal extracts were applied to 6 mm cotton disks at 0.1 mg/disk, then the disks subsequently placed on MRSA-inoculated agar. After 24 hour growth, the *L. persoonii* crude extract displayed a 12 mm diameter (disk plus clear zone) zone of inhibition (ZOI). This strain of MRSA has been found to be broadly resistant to a variety of antimicrobials including: methicillin, centhromycin, azithromycin, erythromycin, clindamycin, ampicillin, chloramphenicol, gentamicin, tetracycline, ciprofloxacin; as well as intermediary resistance to daptomycin, linezolid and vancomycin. Any zone of inhibition past 10 mm at this level, against this strain, is selected for further investigation as it is considerably better than existing antibiotic agents. 

### 2.4. Isolation of Active Compounds

Plugs of SDA containing *L. persoonii* mycelia were removed and used to inoculate 12 × 400 mL Sabouraud dextrose broth (SDB). Mature cultures were frozen, then freeze dried and extracted 3× with methanol, yielding 1.30 g of crude extract. The residue was subjected to normal phase MPLC yielding 6 fractions: A (632 mg), B (24 mg), C (84 mg), D (105 mg), E (248 mg), F (137 mg). These fractions were screened against MRSA and fraction B was found to be active with a 16 mm ZOI. It was further separated on reverse phase HPLC to afford pure cytosporones B (**1**, 1.1 mg), C (**2**, 0.5 mg) and E (**3**, 1.9 mg). The compounds were identified by comparison of their mass and ^1^H and ^13^C NMR spectra to those previously reported [14]. 

### 2.5. Bioactivity of Cytosporones B, C and E

Screening against a highly resistant MRSA strain (USA100) established (Table 1) minimum inhibitory concentration (MIC), minimum bactericidal counts (MBC) and minimum biofilm eradication concentration (MBEC). At MIC, cytosporone B (**1**) demonstrated a 4.2-fold reduction in bacterial viability and at twice the MIC, resulted in complete killing of the bacteria. Furthermore, at MIC, a 2-fold reduction in biofilm formation was observed, and at twice the MIC, 168-fold reduction occurred. At higher concentrations, it appears strongly active toward biofilms, which is uncommon for antibiotics; however, cytosporone B is cytotoxic toward A549 cells (TI_90_ = IC_90_ A549/MIC_90_ = 6). Cytosporone C (**2**) was found to be inactive against MRSA at low µM doses and thus was not tested further. 

**Table 1:** Summary of activity against methicillin-resistant *Staphylococcus aureus* (MRSA) USA100 and cytotoxicity of **1**–**3**.

Compound	MRSA (µM)			A549 (µM)	
	MIC	MBC_90_	MBEC_90_	IC_50_	IC_90_
Cytosporone B (**1**)	78	93	110	170	190
Cytosporone C (**2**)	>358	NT ^a^	NT	690	840
Cytosporone E (**3**)	72	45	39	280	440

^a^ NT: Not tested.

Cytosporone E (**3**) was equipotent against USA100 and methicillin-sensitive *S. aureus* (MSSA) strains (72 µM), indicating the intrinsic drug resistant properties of MRSA strains are not helpful in resisting the action of this cytosporone. In effect, the target of the compound is something that the broadly drug resistant strains have not encountered before and suggestive that there is less likelihood that MRSA strains would develop resistance to it over time. In addition, at MIC, it resulted in >5000-fold reduction in bacterial viability, indicating it is strongly bactericidal, and not just bacteriostatic. The cytosporone E MBC90 is significantly below its MIC, further demonstrating developmental potential. Finally, the MBEC assay showed a 183-fold reduction in bacterial viability at MIC, demonstrating a particularly potent activity for this ultra-resistant strain of MRSA. Cytosporone E is also cytotoxic, but reasonably selective for bacteria relative to mammalian cells (TI_90_ = 10).

Cytosporone B (**1**) and C (**2**) were inactive toward *Plasmodium falciparum* at the levels tested (up to 10 µg/mL). Cytosporone E (**3**), however, displayed an IC_90_ of 13 µM, which represents significant selectivity (TI_90_ = 33) for a moderately potent antimalarial drug. 

### 2.6. Epigenetics Studies

**Figure 2:**
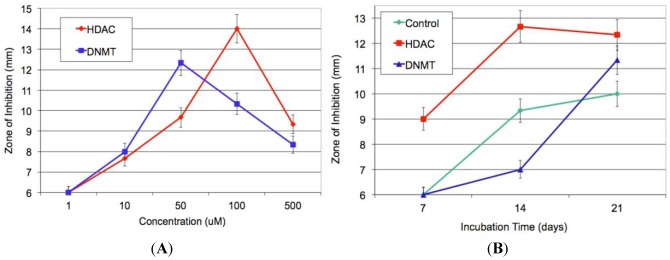
(**A**) Average of triplicate zone of inhibition (ZOI) determinations against MRSA for the crude methanol extract at each concentration of epigenetic modifier; (**B**) Average of triplicate ZOI determinations against MRSA for the crude extract at each time point of incubation. The sterile disks measure 6 mm, thus a result of 6 mm indicates no inhibition at that concentration.

The bioactivity profile of the cytosporones stimulated our interest in titer improvement and generation of additional derivatives, studies we chose to investigate using recently described epigenetic modification [11,12,13]. First, histone deacetylase (HDAC) and DNA methyltransferase (DNMT) inhibitors were employed in dose-response experiments in a 21-day incubation period. Using sodium butyrate as an HDAC inhibitor and 5-azacytidine as a DNMT inhibitor, bioassay results (Figure 2A) revealed the maximal Kirby-Bauer ZOI against USA100 MRSA were obtained using 50 µM DNMT inhibitor and 100 µM HDAC inhibitor. Subsequent time-course experiments, using the previously identified optimal inhibitor concentrations, indicated that the HDAC-inhibited culture exhibited maximal activity as early as week one, while the DNMT-inhibited culture and control required three weeks to achieve maximal activity (Figure 2B). Interestingly, experiments in which we combined the DNMT and HDAC inhibitors produced inconsistent results, with some replicate cultures more active and others inactive. Overall, the best experimental conditions required the HDAC inhibitor and the incubation time of two weeks.

**Figure 3:**
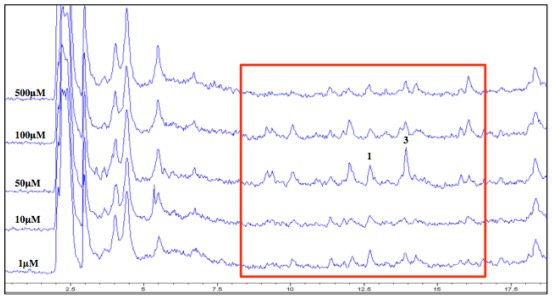
Overlay of LC/MS total ion chromatograms (TIC) from each concentration of crude extracts of DNA methyltransferase (DNMT)-inhibited cultures. Region highlighted indicates varying levels of the peaks of interest.

LC/MS analyses of the culture extracts were found to corroborate the MRSA activity profiles in all cases (Figure 3). For example, in the dose-response experiment for the DNMT inhibitor, several peaks, including those with masses matching cytosporones B (**1**) and E (**3**), in the highlighted region, boast greater areas at the 50 µM concentration *versus* the others. In addition, certain peaks appear to be present in the chromatograms from the 50 and 100 µM DNMT inhibitor that are absent at the other concentrations. This indicates the presence of potential new compounds produced only by the addition of the epigenetic modifier in the fermentation.

### 2.7. Increase in Production and Biological Evaluation of Compounds

Using the optimal growth conditions established, two large-scale cultures were grown; one supplemented with the HDAC inhibitor, and the other, an unmodified control culture. The cultures were grown, extracted and processed side-by-side, in an identical manner, and yielded similar final masses (3.21 g for the modified culture and 2.94 g for the control). Each culture extract was purified first by MPLC, then cytosporone containing fractions subject to HPLC purification. Cytosporones B (**1**), C (**2**) and E (**3**) were the major components in both cultures. However, concentrations were considerably enhanced in the HDAC-inhibited fermentation (Table 2). 

**Table 2:** Yield of Cytosporones from 10 L modified and control cultures.

Compound	Control (mg)	HDAC Inhibited (mg)	Increase (%)
Cytosporone B (**1**)	2.4	8.7	330
Cytosporone C (**2**)	0.7	3.9	510
Cytosporone E (**3**)	3.6	32.1	820

### 2.8. Chemical Analysis of Additional Isolate

**Figure 4:**
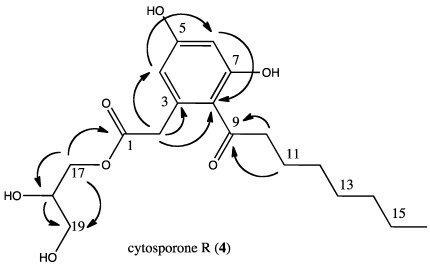
Key HMBC correlations leading to the identification of cytosporone R (**4**).

The HDAC-inhibited fermentation yielded 0.9 mg of a previously undetected cytosporone. Cytosporone R (**4**) exhibited structural features similar to those of cytosporone B (**1**) based on the ^1^H NMR spectrum (Table 3) but the presence of additional proton signals in the δ 3.5–4.5 region, along with MS analysis, indicated an increase in carbon, hydrogen and oxygen count (HR ESI-MS *m/z* 391.1740, Δmmu 0.7 for C_19_H_28_O_7_Na). While some correlations from the 2D NMR experiments identified that the carbon backbone, including the aliphatic side chain, was identical to that of cytosporone B (see Table 2), others helped determine the configuration of the additional atoms in the other side chain. Specifically, the HMBC (Figure 4) correlations from H_2_-17 to C-18 and C-19, both oxygen bearing sp^3^ carbons (δ 71 and 64, respectively) showed the departure from the cytosporone B ethyl ester moiety. In addition, the HMBC correlations from the methine H-18 to both C-17 and C-19 but to no other carbons demonstrated that the side chain ended at C-19 as a methylene with a hydroxyl group. Finally, using the molecular formula determined by HRMS, all that remained was an oxygen and hydrogen, indicating that C-18 bore a hydroxyl group as well. Cytosporone R lacked optical rotation, leading us to believe it is enantiomeric at C_18_ and thus racemic. Cytosporone R was subjected to bioassays against MRSA but found inactive (MIC 503 µM). These experiments cannot clarify whether cytosporone R arose as a response to epigenetic signaling or merely increased sufficiently under epigenetic control to be detected, but nonetheless highlight the utility of the method for drug discovery studies.

**Table 3:** ^1^H and ^13^C NMR spectral data for cytosporone R (**4**) ^a^ and cytosporone B (**1**).

Cytosporone R				Cytosporone B		
Position	δ_C_, mult ^b^	δ_H_ (mult, *J* in Hz)	HMBC	Position	δ_C_	δ_H_
1	173.5, C		2, 17	1	171.6	
2	40.5, CH_2_	3.61, s	4	2	40.1	3.67
3	137.1, C		2	3	137.0	
4	112.0, CH	6.22, d (2.2)	2, 6	4	102.5^c^	6.37
5	161.6, C		4, 6	5	160.7	
6	102.8, CH	6.27, d (2.2)	4	6	111.8^c^	6.31
7	160.0, C		6	7	159.7	
8	121.1, C		4, 6	8	120.8	
9	209.1, C		10, 11	9	206.3	
10	45.2, CH_2_	2.87, m	11, 12, 13	10	44.4	2.90
11	25.6, CH_2_	1.60, br, m	10, 12, 13	11	25.0	1.63
12	30.5, CH_2_	1.25–1.36, br, m	10, 11, 13, 14	12	30.0	1.26–1.32
13	30.3, CH_2_	1.25–1.36, br, m	11, 12, 14, 15	13	29.9	1.26–1.32
14	32.9, CH_2_	1.25–1.36, br, m	12, 13, 15, 16	14	32.5	1.26–1.32
15	23.7, CH_2_	1.25–1.36, br, m	13, 14, 16	15	23.3	1.26–1.32
16	14.4, CH_3_	0.90, t	14, 15	16	14.3	0.88
17	66.8, CH_2_	4.16, dd (11.3, 5.4)	18, 19	17	61.0	4.08
		4.08, dd (11.3, 5.4)	18, 19			
18	71.1, CH	3.84, quin (5.4)	17, 19	18	14.5	
19	64.1, CH_2_	3.54, m	17, 18			

^a^ 500 MHz, CD_3_OD; ^b^ Carbon multiplicity, determined by HSQC; ^c^ Literature values in acetone-*d_6_* [13]. Original publication incorrectly assigned these carbon signals. Shown as they should be, reversed, as described in later publication [19].

## 3. Experimental Section

### 3.1. General Experimental Procedures

Optical rotations were measured on a Rudolph Research Analytical AUTOPOL IV digital polarimeter. IR and UV spectra were measured on Nicolete Avatar 320FT infrared and Hewlett-Packard 8452A diode array instruments, respectively. Medium pressure liquid chromatography was carried out on a Teledyne Isco Combiflash Companion using normal and reverse phase silica Gel and C18 cartridges, respectively, purchased from Teledyne Isco. High performance liquid chromatography was carried out on semipreparative Phenomenex Luna C18(2) reverse phase (250 × 10 mm) and analytical (250 × 4.6 mm) columns using a LC-20A Shimadzu multi-solvent delivery system, a CBM-20A Shimadzu system controller, and a SPD-M20A Shimadzu PDA detector. Low resolution mass spectra were recorded on an Agilent Technologies LC/MSD VL electrospray ionization mass spectrometer. High resolution mass spectra were recorded on an Agilent Technologies LC/MSD TOF electrospray ionization spectrometer. ^1^H and ^13^C NMR spectra were recorded on a Varian Inova instrument operating at 500 MHz for ^1^H, 125 MHz for ^13^C, using residual protonated solvent as ^1^H internal standard or ^13^C absorption lines of solvents for ^13^C internal standard.

### 3.2. Biological Material

A collecting trip into the mangroves of the Florida Everglades was conducted in the summer of 2010. All samples were processed and collected “in-place”, within the guidelines of the State of Florida Department of Environmental Protection, taking precautions from harming these ecologically important trees. To remove epiphytes, a portion of a branch was first treated with 30% sodium hypochlorite for 30 s, rinsed thoroughly with sterile water, then treated with 70% isopropanol for 30 s and finally rinsed with sterile water. Shavings of the bark (each less than 2 mm in length, width or thickness) from the surface-sterilized branch were placed onto a Petri dish containing cornmeal agar made with autoclaved seawater. This was incubated at room temperature for 3 weeks, monitoring the growth of microbes each day. Single filaments of fungi were removed and re-plated repeatedly until pure colonies were obtained.

### 3.3. DNA Extraction

The fungus was grown on a plate of SDA for 7 days. An area of 1 cm^2^ of the fungal mat was cut and placed into a 1.5 mL Eppendorf tube with 600 µL of PBS buffer and about 0.5 cm of 0.1 mm glass disruption beads. The tube was placed into a bead beater and pulsed for 1 m, three times, to lyse the cells. The tubes were centrifuged and the supernatant removed to a clean microfuge snap-cap tube. 200 µL of 1.6% sarkosyl and 25 µg of proteinase K were added, and incubated at 60 °C for 60 m. After incubation, 800 µL of phenol/chloroform was added and the samples vortexed. The resulting emulsion was centrifuged for 5 m and the aqueous layer was removed and placed into a new tube. 500 µL of isopropanol and 100 µL of 3 M sodium acetate were then added to tubes before being mixed. Tubes were then placed at −80 °C for 2 h to allow DNA precipitation. The sample tube was then vortexed for 5 m and the supernatant was discarded. The DNA pellet was washed with 500 µL of 70% ethanol and resuspended in DI water in a refrigerator overnight.

### 3.4. PCR Parameters

DNA from the fungus was amplified in two PCR mixtures containing the following total amounts: 2 µL of DNA, 12.5 µL Taq DNA Polymerase, 6.5 µL of DI water, 2 µL of forward primer nu-SSU-0817-5′ (TTAGCATGGA ATAATRRAATAGGA) and either 2 µL of reverse primer nu-SSU-1196-3′ (TCTGGACCTGGTGAGTTTCC) or nu-SSU-1536-3′ (ATTGCAATGCYCTATCCCCA) [20]. All reagents were combined and heated at 94 °C for 2.5 m. Thirty-five cycles of PCR were then performed by using 94 °C for 0 s, 56 °C for 10 s, and 72 °C for 30 s, followed by 72 °C for 2.5 m.

### 3.5. Gel Step and Isolation of DNA

1% agarose gel was poured into a gel caster with 10 µL of 10 mg/mL solution of ethidium bromide. When solid, it was placed into a gel electrophoresis apparatus filled with TAE buffer (a mixture of Tris base, acetic acid and EDTA). 25 µL of the PCR sample mixtures were added to wells alongside 25 µL of a 1 kb DNA ladder. Samples were then run at 80 volts and 400 mA for 30 m. The resulting gel was viewed under a UV lamp and the portions containing DNA were removed using a surgical blade. The DNA was then isolated and purified using a QIAquick Gel Extraction kit.

### 3.6. Fungus Identification

The DNA was sent to Eurofins MWG Operon for sequencing. Upon receiving the results, they were compared to the BLAST database and the fungus was found to be 99% identical to *Leucostoma persoonii*.

### 3.7. Initial Culture and Screening

The fungi isolated from the expedition were each grown onto two Petri dishes containing SDA at 30 °C for 3 weeks. The resulting cultures were freeze-dried and extracted in methanol. The resulting crude extracts were concentrated, and each was diluted to an approximate concentration of 30 mg/mL in DMSO. The samples were then submitted for screening.

### 3.8. Malaria Assay

Malaria screening was done as previously reported [21].

### 3.9. Methicillin-Resistant Staphylococcus Aureus Strain

The strain of MRSA used in our bioassays is a hospital-associated clone from the CDC USA 100 lineage. It was isolated from Tampa General Hospital and was found to be broadly resistant to a variety of antimicrobials, including: methicillin, centhromycin, azithromycin, erythromycin, clindamycin, ampicillin, chloramphenicol, gentamicin, tetracycline, ciprofloxacin; as well as intermediary resistance to daptomycin, linezolid and vancomycin. It is thus deemed an extensively drug resistant strain, and something of a worst-case scenario MRSA isolate.

### 3.10. Disk Diffusion Assay

Assays for crude extracts and fractions were performed as described previously [22]. 

### 3.11. Natural Product Extraction and Isolation

For the isolation of bioactive natural products, the fungus was grown following the optimal epigenetic modifier conditions identified previously at the larger scale of 10 L. The lyophilized fungus was extracted three times with MeOH. The extract was concentrated to yield a mass of 3.21 g. The residue was subjected to reverse phase MPLC using a gradient (10% MeOH in purified water to 100% MeOH) to give 8 fractions: A (942 mg), B (317 mg), C (209 mg), D (158 mg), E (442 mg), F (268 mg), G (94 mg) and H (85 mg). These fractions were screened against MRSA and fraction E was found to be active with a ZOI of 20 mm. It was further separated using normal phase MPLC using a gradient (100% hexanes to 100% ethyl acetate, then to 100% methanol) to give 16 fractions: A (9 mg), B (96 mg), C (21 mg), D (25 mg), E (13.4 mg), F (20 mg), G (13 mg), H (15 mg), I (24 mg), J (35 mg), K (40 mg), L (32 mg), M (72 mg), N (31 mg), O (53 mg), P (103 mg). The fractions were once again tested against MRSA and the active fraction, B, was further separated on reverse phase HPLC using a gradient (20% acetonitrile in purified water to 100% acetonitrile) to afford pure cytosporones in the chromatographic sequence of R (**4**) E (**1**), C (**3**) and B (**2**). ^1^H and ^13^C NMR spectra of cytosporones B, C and E matched literature values [14].

### 3.12. Microtiter MIC and MBC Determination Assays

The minimum inhibitory concentration (MIC) of the pure compounds was determined as follows. Broth cultures were prepared for *S. aureus* strains as described above. These were diluted 1:1000 in fresh Trypticase Soy Broth (TSB) and 200 µL was applied to the wells of a sterile 96-well plate. Compounds (in DMSO) were added to wells at concentrations ranging from 1, 10, 25, 50 to 100 µg/mL in a volume no greater than 5 µL. Concentrations were adjusted to lower levels if applicable. Samples were mixed by pipetting and the plates were incubated at 37 °C overnight. MICs were determined by visual inspection for the minimum concentration of compound producing no bacterial growth, as determined by a lack of turbidity. Assays were repeated three times in order to verify the MIC determinations. Negative controls performed with DMSO alone revealed no inhibition to bacterial growth. As a positive control, vancomycin was used and was found to have an MIC of 1 µg/mL. Minimum bactericidal concentration (MBC) counts were performed on the MIC cultures by removing the growth media from each well the following morning, serially diluting, and plating onto Trypticase Soy Agar (TSA). These were incubated overnight at 37 °C and the bacterial loads of each well were determined by enumeration. Percentage killing was determined by the comparison of drug-containing wells to control wells where no compound was added.

### 3.13. Biofilm Assay

We performed minimum biofilm eradication count (MBEC) assays on cytosporones to assess their activity towards this form of bacterial growth. These were performed by generating *S. aureus* biofilms in a 96-well microtitre plate, as previously described [23]. Once established, biofilm cultures were exposed to fresh growth media containing a range of concentrations of 15, 20, 25 and 50 µg/mL of the cytosporones. Control analyses were performed in parallel containing no compound. Cultures were incubated, covered, for 24 h at 37 °C without shaking. After this time the culture media was aseptically aspirated, and the wells rinsed twice with PBS to remove non-adherent cells. Fresh PBS (100 µL) was then added to the wells, and mixed by vigorous pipetting, to dislodge the biofilm matrix from the surface of the wells. These samples were serially diluted, plated on TSA, with cells enumerated after overnight incubation at 37 °C. Each of the above analyses was performed with 3 biological replicates, each of which also had 3 technical replicates. By comparing CFU/mL against control, no-exposed cultures, we were able to obtain a quantitative assessment of the impact of cytosporones on *S. aureus* biofilms.

### 3.14. Cytotoxicity Assay

Cell line A549 (adenocarcinomic human alveolar epithelial cells) was cultured in F-12K Nutrient Mixture (Kaighn’s Modification) media containing L-glutamine, supplemented with 10% fetal bovine serum and 1% penicillin-streptomycin. For the assay, the A549 cells were diluted to 1.33 × 10^5^ cells/mL in DMEM F12 media with L-glutamine, without HEPES or phenol red, and supplemented with 2% fetal bovine serum and 1% penicillin-streptomycin. The A549 cells were dispensed into 96 well plates at a volume of 90 µL/well giving a final concentration of 12,000 cells per well. Plates were incubated for 24 h at 37 °C and 5% CO_2_. After 24 h, 10 µL of the test compounds at concentrations of 0.4, 1.2, 3.7, 11, 33.3 and 100 µg/mL were added to the 96 well plates containing A549 cells followed by 72 h of incubation at 37 °C and 5% CO_2_. A Beckman-Coulter Biomek NX 3000 was used to dispense cells and dispense test compounds to the 96 well plates. Positive (bronopol) and negative (blank DMSO) controls were included on each assay plate. After the incubation period, cell proliferation was assessed using Promega’s CellTiter 96 Aqueous One Solution Cell Proliferation Assay reagent. Into each well 20 µL of reagent was added followed by incubation for 3 h at 37 °C and 5% CO_2_. A Biotek plate reader was used to read absorbance at 490 nM and IC_50_ values were determined for each test compound.

### 3.15. Epigenetics Studies

The fungus was subjected to a series of epigenetic modification experiments, including types of modifiers (5-azacytidine as a DNA methyltransferase (DNMT) inhibitor and sodium butyrate as a histone deacetylase (HDAC), or a combination of the two), a modifier concentration gradient of 1, 10, 50, 100 and 500 µM, and a time course (1, 2 and 3 weeks). Culture experiments were performed in triplicate. Plugs of agar containing mycelia from small-scale solid phase cultures of the fungus were placed into vessels containing 125 mL of sterile SDB. The vessels were then placed on a rotary shaker at 20 rpm for 48 h at room temperature. Then an aliquot of the epigenetic modifier was added to reach appropriate final concentrations of 1–500 µM, to achieve desired dose-response concentrations. After modifier treatment, vessels were shaken for an additional 5–19 days, to achieve the desired time course. The resulting cultures were lyophilized and extracted in MeOH. All biological assays and chemical experiments for the epigenetic experiments were performed with these extracts.

### 3.6. Cytosporone R (4)

Colorless oil; [α]^25^_D_ +0 (*c* 0.1, MeOH); UV/Vis (MeOH) λ_max_ (ε): 269 (802), 297 (609); IR (thin film): 3237, 2928, 2855, 1706, 1589, 1467, 1371, 1331, 1269, 1241, 1165, 1111 cm^−1^; HRESIMS *m/z* [M + Na]^+^ 391.1740 (C_19_H_28_O_7_Na calculated 391.1733); ^1^H and ^13^C NMR, see Table 3.

## 4. Conclusions

Cytosporone E (**3**) displays modest potency against both the highly resistant USA100 strain of MRSA, as well as *Plasmodium falciparum*, with sufficiently low mammalian cytotoxicity to provide a therapeutic index >10 for both infectious diseases. Epigenetic modification of fungal cultures demonstrated noteworthy utility in this case, increasing drug titers significantly and providing access to new chemodiversity. 
